# Plasma Biomarkers Discriminate Clinical Forms of Multiple Sclerosis

**DOI:** 10.1371/journal.pone.0128952

**Published:** 2015-06-03

**Authors:** Marta Tejera-Alhambra, Armanda Casrouge, Clara de Andrés, Ansgar Seyfferth, Rocío Ramos-Medina, Bárbara Alonso, Janet Vega, Lidia Fernández-Paredes, Matthew L. Albert, Silvia Sánchez-Ramón

**Affiliations:** 1 Department of Immunology, Hospital General Universitario Gregorio Marañón, Madrid, Spain; 2 Department of Immunology, Center for Human Immunology, Institut Pasteur, Paris, France; 3 Department of Immunology, INSERM U818, Institut Pasteur, Paris, France; 4 Department of Neurology, Hospital General Universitario Gregorio Marañón, Madrid, Spain; 5 STAT-UP Statistical Consulting&Services, Madrid, Spain; 6 Center Alicia Koplowitz for Multiple Sclerosis of the Community of Madrid, Madrid, Spain; 7 Department of Clinical Immunology, Hospital Clínico San Carlos, Madrid, Spain; Hospital General Dr. Manuel Gea González, MEXICO

## Abstract

Multiple sclerosis, the most common cause of neurological disability in young population after trauma, represents a significant public health burden. Current challenges associated with management of multiple sclerosis (MS) patients stem from the lack of biomarkers that might enable stratification of the different clinical forms of MS and thus prompt treatment for those patients with progressive MS, for whom there is currently no therapy available. In the present work we analyzed a set of thirty different plasma cytokines, chemokines and growth factors present in circulation of 129 MS patients with different clinical forms (relapsing remitting, secondary progressive and primary progressive MS) and 53 healthy controls, across two independent cohorts. The set of plasma analytes was quantified with Luminex xMAP technology and their predictive power regarding clinical outcome was evaluated both individually using ROC curves and in combination using logistic regression analysis. Our results from two independent cohorts of MS patients demonstrate that the divergent clinical and histology-based MS forms are associated with distinct profiles of circulating plasma protein biomarkers, with distinct signatures being composed of chemokines and growth/angiogenic factors. With this work, we propose that an evaluation of a set of 4 circulating biomarkers (HGF, Eotaxin/CCL11, EGF and MIP-1β/CCL4) in MS patients might serve as an effective tool in the diagnosis and more personalized therapeutic targeting of MS patients.

## Introduction

Multiple sclerosis (MS), the most common cause of neurological disability in young population after trauma, represents a significant personal, social and economic public health burden. MS is a chronic autoimmune inflammatory disorder of the central nervous system (CNS) characterized by multiple demyelination lesions, axonal degeneration and oligodendrocyte and neuronal loss. The precise etiology of MS remains unknown, although it is widely held that MS is a Th1/Th17 autoimmune disease, where self-reactive effector T cells initiate the inflammatory cascade. The clinical course of MS goes from an early inflammatory phase of the disease with relapses and remissions, where patients may respond to immunomodulatory drugs, to a progressive and neurodegenerative phase that is unresponsive to any currently available treatment. Between 60–70% of patients with a relapsing-remitting MS (RR-MS) form evolve to a secondary-progressive form of MS (SP-MS). About 20% of patients suffer from a progressive onset of the disease without remissions of infaust prognosis, known as primary progressive MS (PP-MS) [1]. In either case, it remains plausible that the various clinical MS forms may represent divergent etiologies, given the distinct pathological patterns and the clinical characteristics they exhibit. Although CNS magnetic resonance imaging (MRI) and the presence of oligoclonal bands in the cerebrospinal fluid (CSF) have helped in the diagnosis of MS, they do not discriminate between the inflammatory and progressive forms at MS.

Many cytokines and their receptors have been predominantly detected in MS lesions and they are thought to play a role in MS pathogenesis via immune system activation as well as via damaging neuronal cells. Proinflammatory cytokines have been extensively studied. CSF levels of proinflammatory cytokines are often elevated in MS patients [2–8].

The current challenges in the management of MS patients are linked to the lack of biomarkers capable of stratifying the different clinical forms of MS. This is a high priority due to the need to define those patients that may evolve to progressive forms. Indeed, the development of minimally invasive biomarkers represents an important avenue for discriminating among the different forms of the disease and for predicting treatment response. They can also help to shed light on MS pathogenesis.

In our cohort of MS patients, we evaluated plasma profiles of cytokines, chemokines and growth factors both individually using ROC curves and in combination using logistic regression analysis for their predictive power regarding the clinical outcome. We have found a set of potential biomarkers differently expressed in the relapsing-remitting MS patients compared to MS patients in the progressive phase of the disease that might serve as an effective tool for stratifying MS patients and better target personalized therapies for this complex disease.

## Materials and Methods

### Subjects

A total of 182 subjects were studied in this work. Among them, 129 patients with definite MS diagnosis according to McDonald’s criteria [9] were consecutively recruited from November 2010 to June 2012, at the Unit of Multiple Sclerosis of the University General Hospital Gregorio Marañón and from Center Alicia Koplowitz for Multiple Sclerosis of the Community of Madrid, Spain. A group of 53 age-matched healthy controls (HC) (29 women/24 men; age: 37 years (31–43) median value (IQR1-3)) from the Blood Donor Bank of the University Hospital Gregorio Marañón was also included. Patients were considered and analyzed as two independent cohorts: the test cohort (cohort 1) was recruited from November 2010 to February 2011 and consisted of 65 MS patients (47 women and 18 men) and 16 HC (9 women and 7 men); the validation cohort (cohort 2) was recruited from June 2011 to June 2012 and consisted of 64 MS patients (38 women and 26 men) and 37 HC (20 women and 17 men).

MS patients were characterized into two main groups, as “inflammatory MS or RR-MS” and “progressive MS”. The “inflammatory MS/ RR-MS” group consisted of patients diagnosed as Relapsing-Remitting form that were in the inflammatory phase of the disease and was composed of the following subgroups: Recurrent-Remitting MS patients in remission (RR-MS Remission), RR-MS patients with active disease (RR-MS Active), patients at clinical exacerbation or relapse (RELAPSES), RR-MS patients under treatment with IFN-β for a minimum of 6 months who had a good clinical response to IFN-β and were defined as long-term IFN-β responders (RESPONDERS), RR-MS patients that had not responded to IFN-β (NON-RESPONDERS) and were going to start other disease-modifying therapy (DMT). The “progressive MS” group included patients diagnosed as Secondary-Progressive MS (SP-MS) or Primary-Progressive MS (PP-MS). Except for the long-term IFN-β RESPONDERS group, the rest of the patients had not received any glucocorticoid treatment, immunosuppressive or DMT before blood sampling in the three months prior to study entry. The different groups are depicted in Table 1. In the group of RR-MS Remission, there were 17 benign forms of MS that had been symptom-free for years. A patient with RR-MS with active disease (RR-MS Active) was defined as a patient with clinical and paraclinical activity of the disease, that was studied at least 1 month after the end of a clinical exacerbation and was eligible to start a DMT. Relapse was defined as the appearance or reappearance of one or more neurological abnormalities that persisted for at least 24 h and which had been preceded by at least 30 days of stable or improved neurological state, without any underlying infectious disease; blood samples were drawn from relapse patients before initiation of glucocorticoid treatment. Clinical disease severity and disability was rated according to the Kurtzke Expanded Disability Status Scale (EDSS) [10]. For detailed patient characteristics, see Table 1. This study was approved by University Hospital Gregorio Marañón Ethics Committee in Madrid and all subjects enrolled in the study gave written informed consent.

**Table 1:** Epidemiological and clinical characteristics of multiple sclerosis patients included in the study.

		Inflammatory MS/ RR-MS					Progressive MS	
Characteristics	CD-MS	RR-MS Remission	RR-MS Active	RELAPSES	RESPONDERS	NON RESPONDERS	SP-MS	PP-MS
No. of patients^a^	129	23	13	11	20	13	31	18
Gender F/M^a^	85/44	17/6	8/5	8/3	10/10	11/2	22/9	9/9
Age (years)^b^	42 (33–53)	38 (32–46)	29 (26–37)	30 (26–42)	37 (33–41)	38 (29–43)	53 (46–58)	58 (45–64)
Disease duration (yrs)^b^	11 (3–17.5)	9 (5–16)	1 (1–4.5)	1 (0–9)	9 (1.8–16.3)	11 (5–15.5)	19 (13–27)	20.5 (8.8–26.3)
Age at onset (yrs)^b^	28 (24–36)	28 (22–32)	26 (25–35)	28 (25–40)	26 (23–35)	26 (24–38)	32 (26–41)	40 (33–50)
EDSS^b^	2.5 (1.0–5.5)	0.0 (0.0–1.5)	2.5 (1.0–3.0)	3.0 (2.0–4.0)	1.0 (0.0–2.0)	3.5 (2.3–4.5)	8.0 (7.5–8.5)	8.0 (7.3–8.0)
Progression Index^c^	0.3 (0.1–0.6)	0.0 (0.0–0.1)	1.5 (0.2–2.9)	0.8 (0.2–5.4)	0.1 (0.0–0.3)	0.3 (0.2–0.5)	0.4 (0.3–0.6)	0.4 (0.3–0.9)
No. of relapses^d^								
0	73	17	0	6	2	0	30	18
1	18	4	3	3	4	3	1	0
2–5	38	2	10	2	14	10	0	0
Treatment, IFNβ^a^	20	_	_	_	20	_	_	_
Treatment duration (mo)^b^	18 (6–24)	_	_	_	18 (6–24)	_	_	_

CD-MS clinically definite MS group, RR-MS Remission: relapsing remitting MS in remission, RR-MS Active: relapsing remitting MS with active disease, RESPONDERS: RR-MS under treatment with IFNβ, NON-RESPONDERS: relapsing remitting MS that did not respond to Interferon-β, SP-MS: secondary progressive MS, PP-MS: primary progressive MS.

EDSS expanded disability status scale, IFNβ: interferon-β.

^a^ Number of patients;

^b^ Median (25th-75th percentiles)

^c^ EDSS/Disease duration (years);

^d^ Number of relapses during preceding 2 years.

### Multiplexed biomarkers detection

Peripheral blood samples were taken by venous puncture and collected in sterile EDTA Vacutainers, between 8:00 and 10:00 a.m., they were processed immediately or within 2 hours after extraction. Plasma samples were obtained after high speed centrifugation for 10 minutes at 3,500–4,000 rpm and immediately aliquoted and frozen at -80°C for its conservation.

Plasma samples were clarified by high-speed centrifugation and analyzed using Luminex xMAP technology platform. The current investigation required the assembly of an extensive multiplex array consisting of cytokines, chemokines, soluble receptors, growth and angiogenic factors, which were evaluated using bead-based immunoassays. The selected array was the Human cytokine 30-Plex panel (Invitrogen) with the following analytes: IL-1β, IL-1RA, IL-2, IL-2R, IL-4, IL-5, IL-6, IL-7, IL-8, IL-10, IL-12, IL-13, IL-15, IL-17, TNF-α, IFN-α, IFN-γ, GM-CSF, MIP-1α, MIP-1β/CCL4, IP-10, MIG, Eotaxin/CCL11, Rantes/CCL5, MCP-1/CCL2, VEGF, G-CSF, EGF, FGF-basic, and HGF.

### Statistical analysis

Descriptive data are presented as mean ± standard deviation (SD) and median (interquartile range). We compared categorical by the chi-square χ2 test. When multiple groups with continuous outcomes were compared, the nonparametric Kruskal-Wallis rank sum test was used, followed by pairwise Mann-Whitney tests if the former indicated significant differences. Correlations were assessed using Spearman correlation (r_s_) coefficients. Receiver operating characteristic (ROC) curves were used to select the optimal cut-off values of significant variables for considering negative or positive predictors of the development of any given clinical condition studied. Logistic regression and the area under the receiver operating curves (AUROC) were used to determine the diagnostic accuracy of a model that included biomarkers able to discriminate between patients and healthy controls. Data were analyzed with SPSS (Chicago, Illinois) and GraphPad Prism software (CA, USA). A p-value less than 0.05 was considered as statistically significant. The Bonferroni correction was applied to compensate the multiplicity of the tests.

## Results

### Demographic and clinical characteristics of MS patients

In our MS study population, 80 out of 129 (62%) MS patients were classified in the inflammatory phase of the disease and were diagnosed as relapsing-remitting MS: RR-MS Remission n = 26, RR-MS Active n = 13, RESPONDERS n = 20, NON RESPONDERS n = 13 and RELAPSES n = 11 (for 6 out of 11 it was their first clinical episode (CIS), and they were later diagnosed as RR-MS). There were 49 patients in the progressive phase of the disease, 31 were diagnosed as SP-MS and 18 as PP-MS.

RR-MS patients and patients at relapse had similar ages, while progressive patients SP-MS and PP-MS were around 20 years older (Table 1). The lowest disease duration (median 1 year) was in the groups of RR-MS with active disease and patients at relapse. In RR-MS at remission and responders and non-responders to IFNβ, mean disease duration was about a decade and in the progressive forms (SP-MS and PP-MS) two decades. SP-MS and PP-MS had the highest EDSS compared to the other groups. EDSS in RR-MS Remission and in IFNβ RESPONDERS were the lowest (median, 0 and 1, respectively), as expected. The progression index (EDSS/Disease duration (years) was the highest in RR-MS Active patients, as they had been recently diagnosed and presented with considerable disability. As depicted in Table 1, the patients with highest number of relapses in the preceding two years where those under treatment with IFNβ (RESPONDERS) or about to start therapy with a DMT (RR-MS Active and NON RESPONDERS).

### Panel of Plasma Biomarkers that Discriminate between Relapsing-Remitting and Progressive Clinical Forms of Multiple Sclerosis

We proceeded from the hypothesis that biomarkers present in the circulation of patients diagnosed with different clinical forms of MS can provide clinically relevant information pertaining to the progression of the disease and also a basis for the discrimination between the three conditions (RR-MS, SP-MS and PP-MS). The evaluation of this hypothesis was conducted according to the following objectives: firstly, biomarkers levels present in the plasma of several distinct clinical forms of MS were examined in order to identify alterations associated with specific disease forms. Secondly, we focused on the use of a combination of biomarkers using logistic regression for their predictive power achieving superior sensitivity and specificity.

Given the intrinsic heterogeneity of MS, the two cohorts were pooled to increase the number of cases and controls. With all the MS patients globally considered, the 30 analytes were tested as predictors for the discrimination between Relapsing-Remitting and Progressive Clinical forms of MS by ROC curves. For six of them (HGF, Eotaxin/CCL11, MCP-1/CCL2, Rantes/CCL5, EGF and MIP-1β/CCL4) a p-Value < 0.05 was found, which in all six cases was also < 0.05/30 (Table 2). Hence these results are still significant even when applying the Bonferroni correction to the significance threshold, the most conservative method to compensate the multiplicity of the tests.

**Table 2:** Biomarkers that discriminate between Relapsing-Remitting and Progressive Clinical forms of MS by ROC curves.

RR-MS vs Progressive MS	AUC	(95% CI)	p value	Cut off (pg/ml)
**HGF**	0.702	(0.604–0.799)	<0.0002	387
**Eotaxin/CCL11**	0.755	(0.662–0.849)	<0.0001	132
**MCP-1/CCL2**	0.721	(0.626–0.816)	<0.0001	550
**Rantes/CCL5**	0.792	(0.707–0.876)	<0.0001	5536
**EGF**	0.689	(0.592–0.786)	0.0005	66
**MIP-1β/CCL4**	0.702	(0.607–0.798)	<0.0002	109

The areas under the curve (AUC), 95% Confidence Interval (95% CI) and p value for each analyte are depicted in Table 2.

### HGF, Eotaxin/CCL11, MCP-1/CCL2 and Rantes/CCL5 are present in lower concentrations in the plasma of Relapsing-Remitting than in Progressive Multiple Sclerosis patients

The analytes HGF, Eotaxin/CCL11, MCP-1/CCL2 and Rantes/CCL5 were present at significantly lower circulating levels in the RR-MS patients than in the progressive clinical forms of MS patients (SP-MS and PP-MS).

The analysis of the test cohort revealed that HGF, Eotaxin/CCL11, MCP-1/CCL2, Rantes/CCL5 were present in significantly lower concentrations in the inflammatory (RR-MS) phase with respect to the progressive MS clinical forms (SP-MS and PP-MS), findings that were confirmed in the validation cohort (Table 3). HGF, Eotaxin/CCL11 and Rantes/CCL5 differences between these two groups were statistically significant in both cohorts analyzed independently (Table 3). Plasma MCP-1/CCL2 levels in RR-MS patients showed a trend to be lower in the test cohort (p = 0.06), while in the validation cohort, this difference was statistically significant (p = 0.0002).

**Table 3:** HGF, Eotaxin/CCL11, MCP-1/CCL2, Rantes/CCL5, EGF and MIP-1β/CCL4 comparison in the test and validation cohorts.

	RR-MS	Progressive MS
**Test cohort**		
**HGF** [pg/mL]	117 (67–351)**	406 (188–508)
**Eotaxin/CCL11** [pg/mL]	69 (44–99)**	119±65
**MCP-1/CCL2** [pg/mL]	325 (265–519)^#^	447 (337–561)
**Rantes/CCL5** [pg/mL]	2,359 (1,105–6,066)***	7,791 (5,548–7,791)
**EGF** [pg/mL]	55 (8–89)***	8 (8–8)
**MIP-1β/CCL4** [pg/mL]	135 (88–183)***	51 (37–93)
**Validation cohort**		
**HGF** [pg/mL]	334 (239–430)**	536 (344–805)
**Eotaxin/CCL11** [pg/mL]	88 (60–122)***	230 (121–381)
**MCP-1/CCL2** [pg/mL]	357 (205–461)***	680 (391–1,005)
**Rantes/CCL5** [pg/mL]	2,641 (1,649–4,841)***	23,644 (4,029–23,644)
**EGF** [pg/mL]	106 (63–155)^#^	64 (46–106)
**MIP-1β/CCL4** [pg/mL]	131 (95–189)^#^	94 (79–129)

Mann—Whitney statistical test was used for calculation of the reported p-value between RR-MS vs Progressive MS; RR-MS = Relapsing-Remitting MS patients. Note that the Relapsing-remitting group comprises the clinical groups: RR-MS Remission, RR-MS Active, RESPONDERS, NON RESPONDERS and RELAPSES. The Progressive group comprises Secondary (SP-MS) and Primary-progressive patients.

Results are given as Median value (IQR1–3). Statistical significance is marked as:

*p<0.05;

**p<0.01;

***p<0.001.

^#^ p = 0.06 (trend).

**Fig 1:**
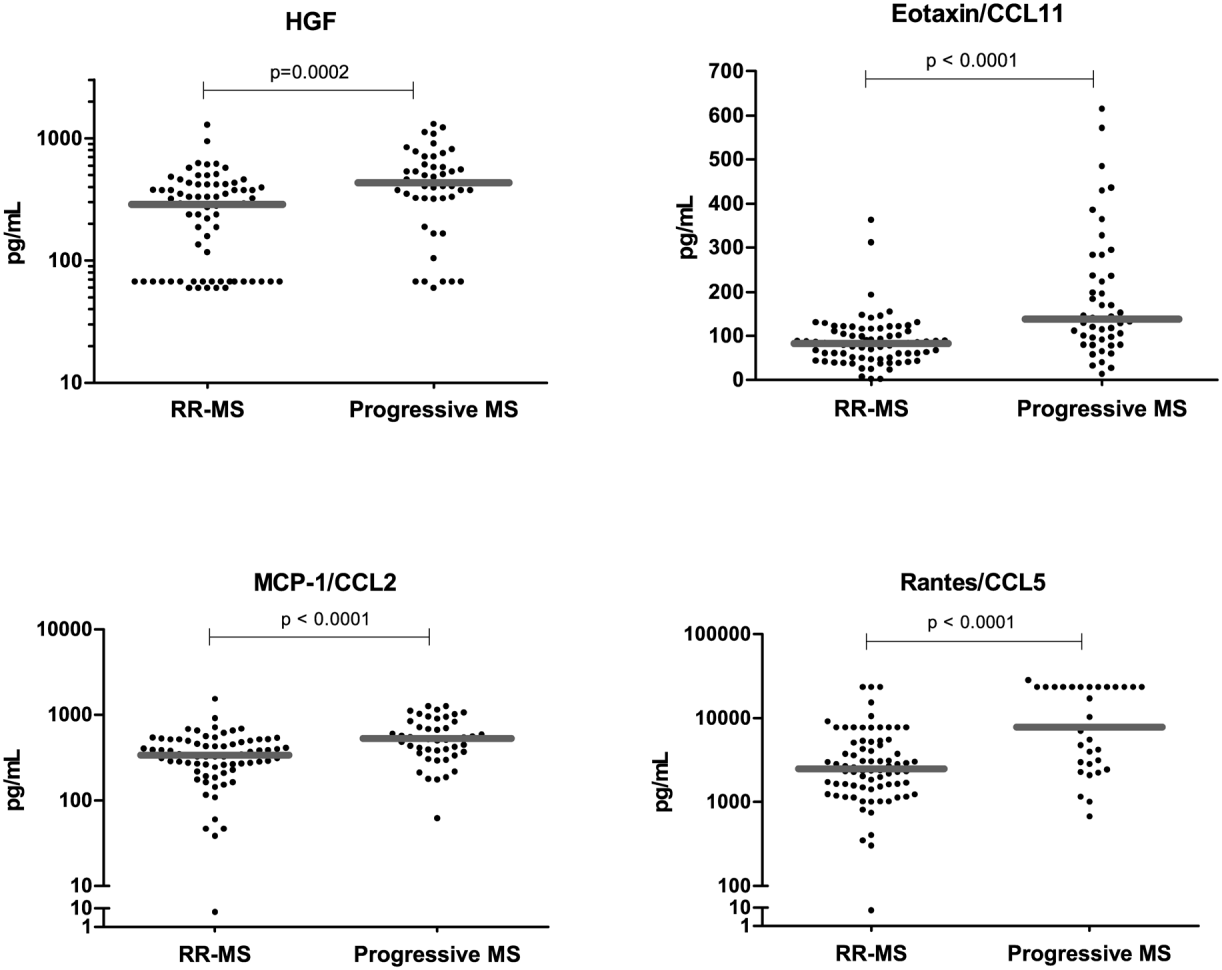
Different plasma levels of HGF, Eotaxin/CCL11, MCP-1/CCL2 and Rantes/CCL5 in MS clinical forms. Plasma levels of HGF, Eotaxin/CCL11, MCP-1/CCL2 and Rantes/CCL5 were lower in relapsing-remitting MS than in the progressive MS patients. Comparison of the plasma levels of HGF, Eotaxin/CCL11 (top dot plots), MCP-1/CCL2 and Rantes/CCL5 (lower dot plots) in relapsing-remitting (RR-MS, n = 80) and in the group of progressive MS (n = 49) patients. Mann—Whitney statistical test was used for calculation of the reported p-value; median values are represented by a gray bar; individual dots indicate single donor values. Note that the Relapsing-remitting group comprises the clinical groups: RR-MS Remission, RR-MS Active, RESPONDERS, NON RESPONDERS and RELAPSES. The Progressive group comprises Secondary (SP-MS) and Primary-progressive patients.

The two cohorts were pooled to increase the number of cases and controls. Again, in MS patients globally considered, we found that plasma levels of HGF, Eotaxin/CCL11, MCP-1/CCL2 and Rantes/CCL5 were significantly lower in the inflammatory (RR-MS patients) than in the progressive forms (SP-MS and PP-MS). In Fig 1 the results from RR-MS are compared to those of the progressive MS patients: (HGF: 294±221 vs 484±310, p = 0.0002; Eotaxin/CCL11: 87±56 vs 187±143, p<0.0001; MCP-1/CCL2: 375±227 vs 588±309, p<0.0001; Rantes/CCL5: 4,173±4,855 vs 11,371±8,800, p<0.0001). Interestingly, the levels of HGF, MCP-1/CCL2 and Eotaxin/CCL11 in the progressive MS patients were similar to those observed in healthy controls (HGF: 484±310 vs 476±652; Eotaxin/CCL11: 187±143 vs 147±138; MCP-1/CCL2: 588±309 vs 538±315), while Rantes/CCL5 was more elevated in the progressive forms with respect to healthy controls (Rantes/CCL5: 11,371±8,800 vs 5,518±6,796). RR-MS patients had significantly lower levels of Eotaxin/CCL11, MCP-1/CCL2 and Rantes/CCL5 than healthy controls (Eotaxin/CCL11: 87±56 vs 147±138, p = 0.001, MCP-1/CCL2: 375±227 vs 538±315, p = 0.001 and Rantes/CCL5: 4,173±4,855 vs 5,518±6,796, p = 0.0002).

The ROC analyses indicated a good performance of plasma HGF, Eotaxin/CCL11, MCP-1/CCL2 and Rantes/CCL5 for the discrimination between RR-MS from progressive clinical forms (Table 2).

HGF correlated strongly with MCP-1/CCL2 both in MS patients and in healthy controls (r_s_ = 0.4674, p<0.0001), whilst HGF correlated with Eotaxin/CCL11 (r_s_ = 0.5574, p<0.0001) and Rantes/CCL5 (r_s_ = 0.3317, p = 0.0002) only in MS patients.

HGF, MCP-1/CCL2, Eotaxin/CCL11 and Rantes/CCL5 correlated also with neurological disability measured by the EDSS: HGF (r_s_ = 0.3254, p = 0.0003), MCP-1/CCL2 (r_s_ = 0.2714, p = 0.003), Eotaxin/CCL11 (r_s_ = 0.3610, p<0.0001), Rantes/CCL5 (r_s_ = 0.4386, p<0.0001).

### EGF and MIP-1β/CCL4 are diminished in Progressive MS clinical forms

Both in the test cohort and in the validation cohort, EGF and MIP-1β/CCL4 plasma levels were lower in the progressive MS forms (SP-MS and PP-MS) with respect to RR-MS patients. In the test cohort, plasma EGF and MIP-1β/CCL4 statistically differed in both classification groups (p<0.0001 and p = 0.0002, respectively); in the validation cohort, both analytes showed a trend (p = 0.06) to be at lower concentrations in the plasma of progressive patients (SP-MS and PP-MS) than in RR-MS patients (Table 3).

**Fig 2:**
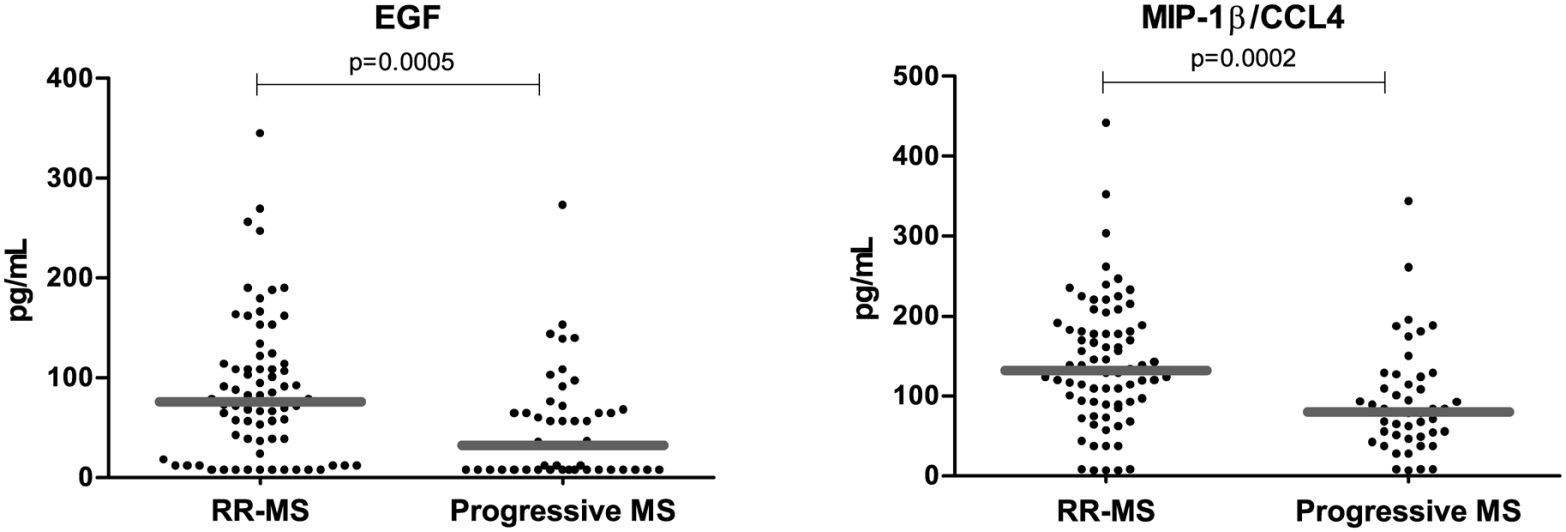
Different plasma levels of EGF and MIP-1β/CCL4 in MS clinical forms. Plasma levels of EGF and MIP-1β/CCL4 were lower in the progressive MS than in relapsing-remitting MS patients. Comparison of the plasma levels of EGF and MIP-1β/CCL4 between patients with relapsing-remitting (RR-MS, n = 80) and in progressive MS patients (n = 49). Mann—Whitney statistical test was used for calculation of the reported p-value; median values are represented by a gray bar; individual dots indicate single donor values. Note that the Relapsing-remitting group comprises the clinical groups: RR-MS Remission, RR-MS Active, RESPONDERS, NON RESPONDERS and RELAPSES. The Progressive group comprises Secondary (SP-MS) and Primary-progressive patients.

The pool of both cohorts displayed the same distribution with significantly diminished circulating levels of EGF and of the chemokine MIP-1β/CCL4 in patients with progressive forms of MS than in RR-MS patients (EGF: 87±71 vs 50±54, p = 0.0005; MIP-1β/CCL4: 141±79 vs 93±67, p = 0.0002) (Fig 2). RR-MS patients and healthy controls showed similar circulating levels of EGF and MIP-1β/CCL4 (EGF: 87±71 vs 115±92; MIP-1β/CCL4: 141±79 vs 170±116) and progressive patients had significantly lower levels of EGF and MIP-1β/CLL4 than HC (EGF: 50±54 vs 115±92, p<0.0001 and MIP-1β/CCL4: 93±67 vs 170±116, p = 0.0002, respectively). The ROC curves of plasma EGF and MIP-1β/CCL4 were used to assess the potential identification between RR-MS and progressive (SP-MS or PP-MS) clinical forms (Table 2).

As expected, EGF and MIP-1β/CCL4 correlated negatively with the disability score EDSS:

EGF (r_s_ = -0.2906, p<0.002, MIP-1β/CCL4 (r_s_ = -0.355, p<0.0001).

### Logistic regression

We carried out the multivariate logistic regression analysis including all these six analytes (HGF, MCP-1/CCL2, Eotaxin/CCL11, Rantes/CCL5, EGF and MIP-1β/CCL4) as independent variables and the clinical MS-forms (Relapsing-Remitting vs Progressive) as the dichotomous target variable. We obtained a model that included the 4 analytes HGF, EGF, Eotaxin/CCL11 and MIP-1 β/CCL4) (Table 4) as predictors for the MS-clinical form, while Rantes/CCL5 and MCP-1/CCL2 did not reach statistical significance to be included.

**Table 4:** Logistic regression with HGF, Eotaxin/CCL11, EGF and MIP-1β/CCL4 as independent variables and the clinical MS-forms (Relapsing-Remitting vs Progressive MS) as the dichotomous target variable.

RR-MS vs Progressive MS	P value	O.R.	C.I. (95%)
**HGF** [pg/mL]	0.0085	1.0038	1.0010–1.0066
**Eotaxin/CCL11** [pg/mL]	0.0004	1.0147	1.0066–1.0229
**EGF** [pg/mL]	0.0056	0.9808	0.9675–0.9944
**MIP-1β/CCL4** [pg/mL]	0.0171	0.9873	0.9769–0.9977
Constant	0.1049	0.4021	

O.R. (odds ratio); C.I. (95%) Interval of confidence.

With this combination model, we were able to give the odds for a specific patient to be diagnosed as progressive MS, i.e. the probability—P—of being classified as progressive clinical form of MS divided by the probability of developing a relapsing-remitting form 1-P, according to the model are given by the product of 0.4021 and the four odds ratios each raised by the respective predictors’ values (in pg/mL) measured in an individual patient. Each analyte has an odds ratio (O.R.) which is raised to the power of its respective value [pg/mL] in a patient. For instance, for a patient with values [pg/mL] of Eotaxin/CCL11: 200; HGF: 323; EGF: 17; MIP-1β/CCL4: 54, the odds are 0.4021 · 1.0147^200^ · 1.0038^323^ · 0.9808^17^ · 0.9873^54^ = 9.14. Hence, the model assigns to this patient a probability of having a progressive form of 9.14 / (1+9.14) = 90.1%.

HGF and Eotaxin/CCL11 with O.R. >1 (Table 4) are risk factors for developing a progressive clinical form of MS, while EGF and MIP-1β/CCL4 with O.R. <1 are protective factors for developing a progressive clinical form of MS.

Considering odds >/< 1, i.e. a greater/less probability of having a progressive versus RR-MS clinical form according to the model, as a positive/negative prognosis, the model gives a sensitivity of 71.7%, a specificity of 89.9%, a positive predictive value (PPV) of 82.5%, a negative predictive value of 82.7% and an accuracy of 82.6% for our cohort of MS patients.

Each of the analytes included in the combination model by multivariate logistic regression analysis was independently analyzed by univariate regression analysis (ROC Analysis) for its predictive ability to discriminate between a Progressive and a RR-MS patient. For each analyte, an specific cut-off value: HGF >387, Eotaxin/CCL11 >132, EGF <66 and MIP-1β/CCL4 <109 pg/ml could discriminate with different sensitivity, specificity, positive predictive value (PPV), negative predictive value (NPV) and accuracy between a Progressive and a RR-MS patient. The combination of the four plasma levels of HGF, Eotaxin/CCL11, EGF and MIP-1β/CCL4 with multivariate logistic regression analyses gave a higher sensitivity and specificity (Table 5).

**Table 5:** Clinical utility of HGF, Eotaxin/CCL11, EGF and MIP-1β/CCL4 as MS biomarkers and clinical utlity of their combination in a model by multivariate logistic regression analysis.

RR-MS vs Progressive MS	Sensitivity	Specificity	PPV	NPV	Accuracy
**HGF**	59.6%	72.6%	58.3%	73.6%	67.5%
**Eotaxin/CCL11**	53.2%	90.8%	78.1%	75.8%	76.4%
**EGF**	74.5%	59.7%	54.7%	78.2%	65.5%
**MIP-1β/CCL4**	70.2%	68.4%	57.9%	78.8%	69.1%
**Model by logistic regression analysis**	71.7%	89.9%	82.5%	82.7%	82.6%

PPV: positive predictive value, NPV: negative predictive value.

### FGFb can discriminate between Primary and Secondary Progressive patients and between Primary Progressive patients and Relapsing-Remitting patients undergoing clinical relapse

**Fig 3:**
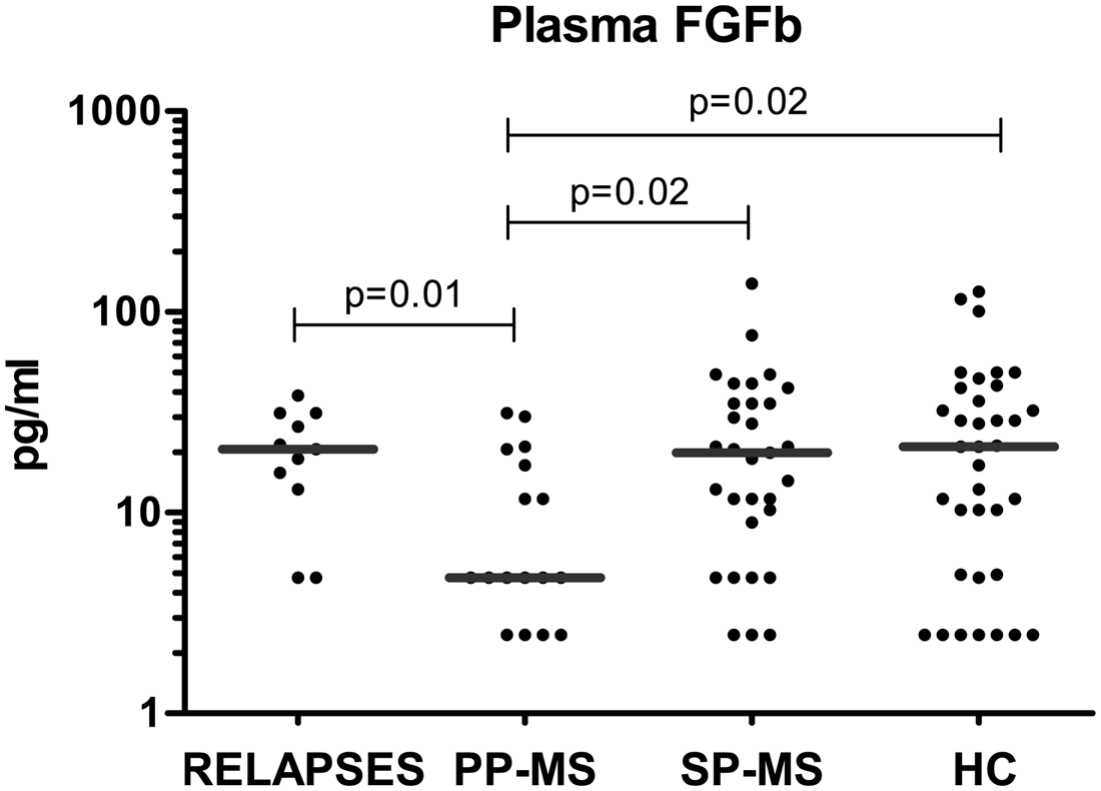
FGFb levels were significantly decreased in primary progressive MS patients. Comparison of the plasma levels of FGFb between primary progressive MS patients (PP-MS, n = 18) with respect to healthy controls (HC, n = 36), to secondary progressive MS patients (SP-MS, n = 31) and to patients undergoing a clinical relapse (RELAPSES, n = 11). Mann—Whitney statistical test was used for calculation of the reported p-value; median values are represented by a gray bar; individual dots indicate single donor values.

Plasma FGFb was markedly diminished in PP-MS patients when compared to SP-MS (11±10 vs 26±27, p = 0.01), patients undergoing a clinical relapse (11±10 vs 21±11, p = 0.01), or healthy controls (11±10 vs 28±31, p = 0.02) (Fig 3). This distinction is important because MS at onset can be divided into two main forms: relapsing-remitting and primary progressive.

### VEGF Levels were higher in Secondary Progressive than in Relapsing-Remitting MS

**Fig 4:**
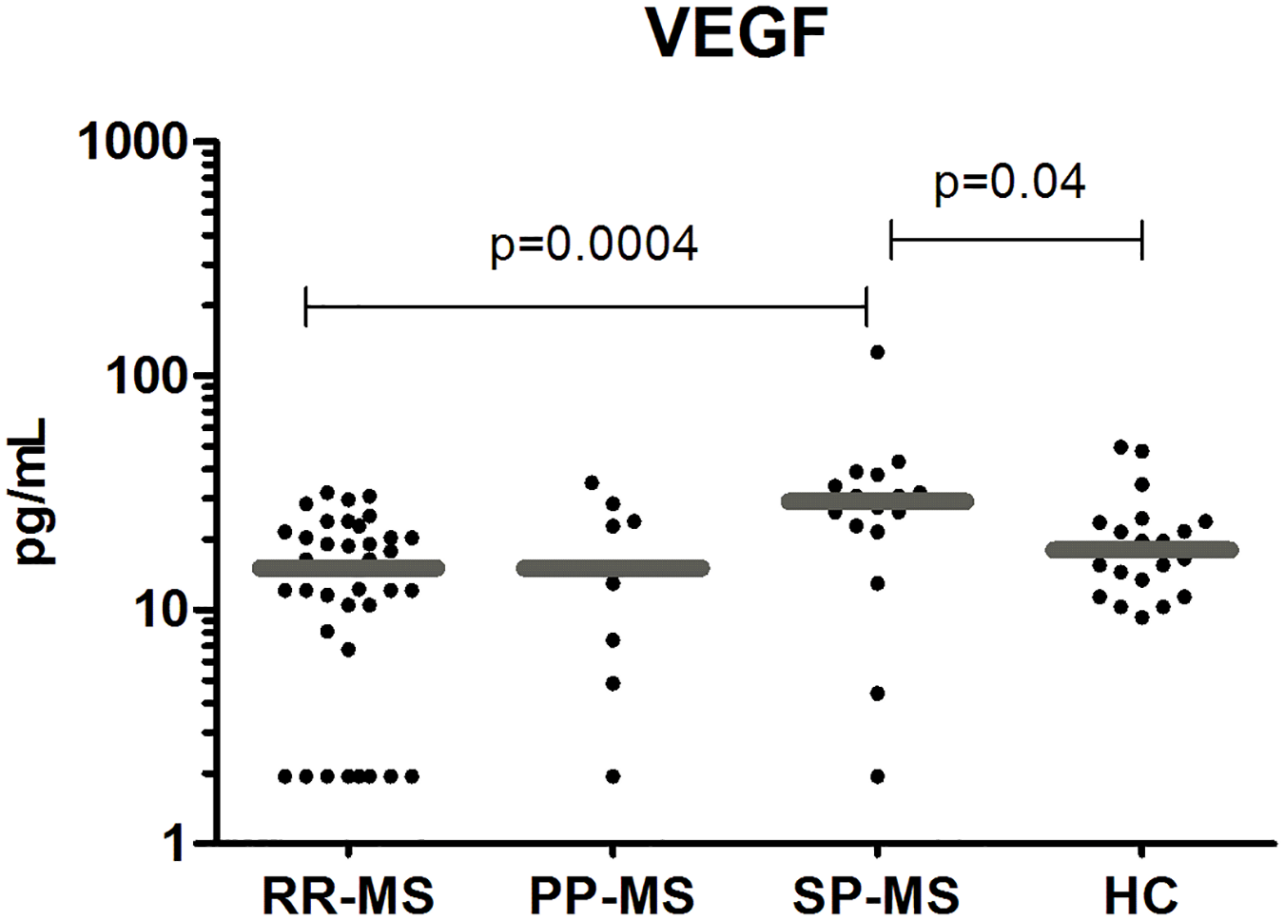
VEGF levels were significantly higher in secondary progressive MS patients. Plasma levels of the vascular growth factor (VEGF) in the validation cohort, were higher in secondary progressive MS patients (SP-MS, n = 16) than in patients with the relapsing-remitting form (RR-MS, n = 38) and than in healthy controls (HC, n = 20). The SP-MS patients had higher plasma levels of VEGF than primary progressive MS patients (PP-MS, n = 9) but this difference did not reach statistical significance. Mann—Whitney statistical test was used for calculation of the reported p-value; median values are represented by a gray bar; individual dots indicate single donor values.

Patients with SP-MS had significantly higher circulating levels of the vascular epidermal growth factor (VEGF) than all the patients with RR-MS (32±28 vs 15±9, p = 0.0004) and than healthy controls (32±28 vs 21±11, p = 0.04) (Fig 4). Patients with PP-MS had also lower circulating levels of VEGF than SP-MS, but this difference was not statistically significant.

### IP10, MCP-1/CCL2 circulating levels are significantly higher in Relapsing-Remitting patients that respond to IFN-β treatment than in Non Responders

**Fig 5:**
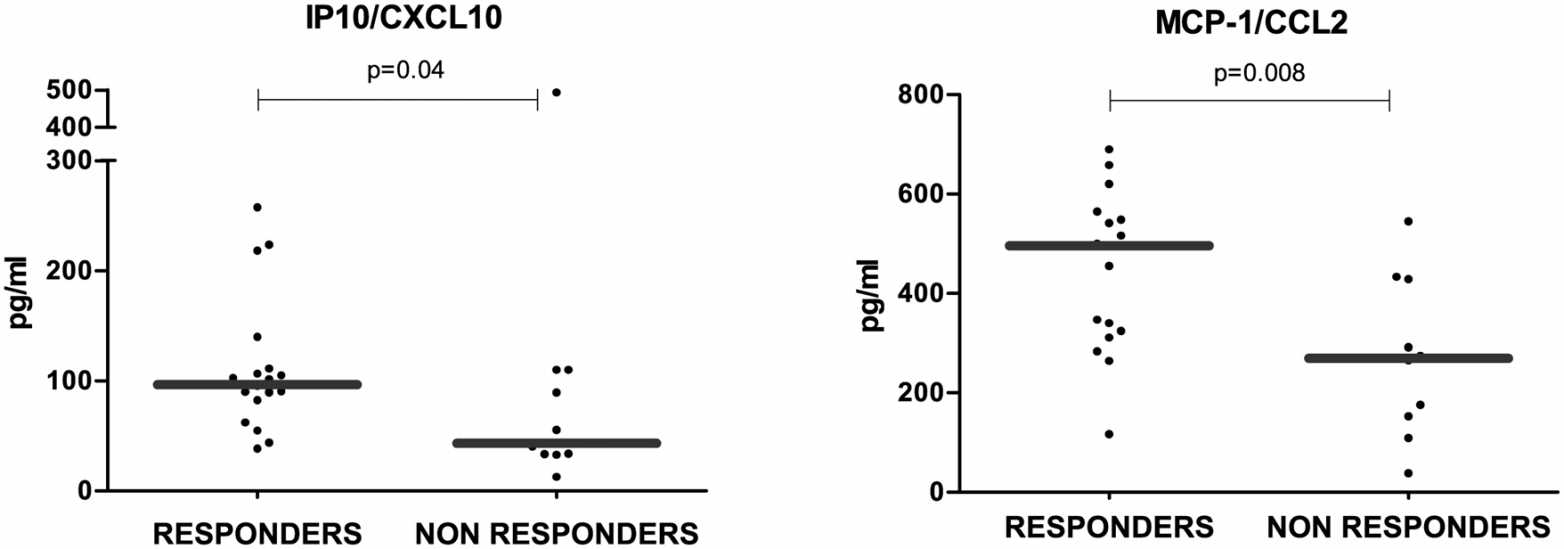
IP10/CXCL10 and MCP-1/CCL2 levels were higher in MS patients with good response to IFN-β treatment. Long-term IFN-β treated RR-MS patients (RESPONDERS, n = 20) presented with higher circulating levels of IP10/CXCL10 and MCP-1/CCL2 than non responders to IFN-β (NON RESPONDERS, n = 13). Mann—Whitney statistical test was used for calculation of the reported p-value; median values are represented by a gray bar; individual dots indicate single donor values.

Among the chemokines tested, IP10/CXCL10 and MCP-1/CCL2 were significantly higher in RR-MS patients under treatment with IFN-β (RESPONDERS) than in those patients who had been previously treated with IFN-β and did not respond to therapy and had clinical activity (NON RESPONDERS) (IP10: 111±60 vs 96±136, p = 0.04; MCP-1/CCL2: 446±158 vs 272±152, p = 0.008) (Fig 5). These two cytokines are induced by type I IFN, but only IP10/CXCL10 levels were significantly higher in the RESPONDERS group when compared to the levels of the group of HC (111±60 vs 86±86, p = 0.005) and to those levels of the rest of RR-MS patients without treatment (RR-MS Remission, RR-MS Active, RELAPSES and NON RESPONDERS) (111±60 vs 75±78, p = 0.0003). IP10 levels were also higher in the progressive forms of MS (SP-MS and PP-MS) than in the rest of RR-MS patients without treatment studied: In PP-MS patients, there was a modest increase (96±75 vs 75±78, p = 0.05), whereas in SP-MS patients these differences were more remarkable (102±52 vs 75±78, p = 0.0001). SP-MS had also higher circulating levels of IP10 than the group of healthy controls (102±52 vs 86±86, p = 0.01).

**Fig 6:**
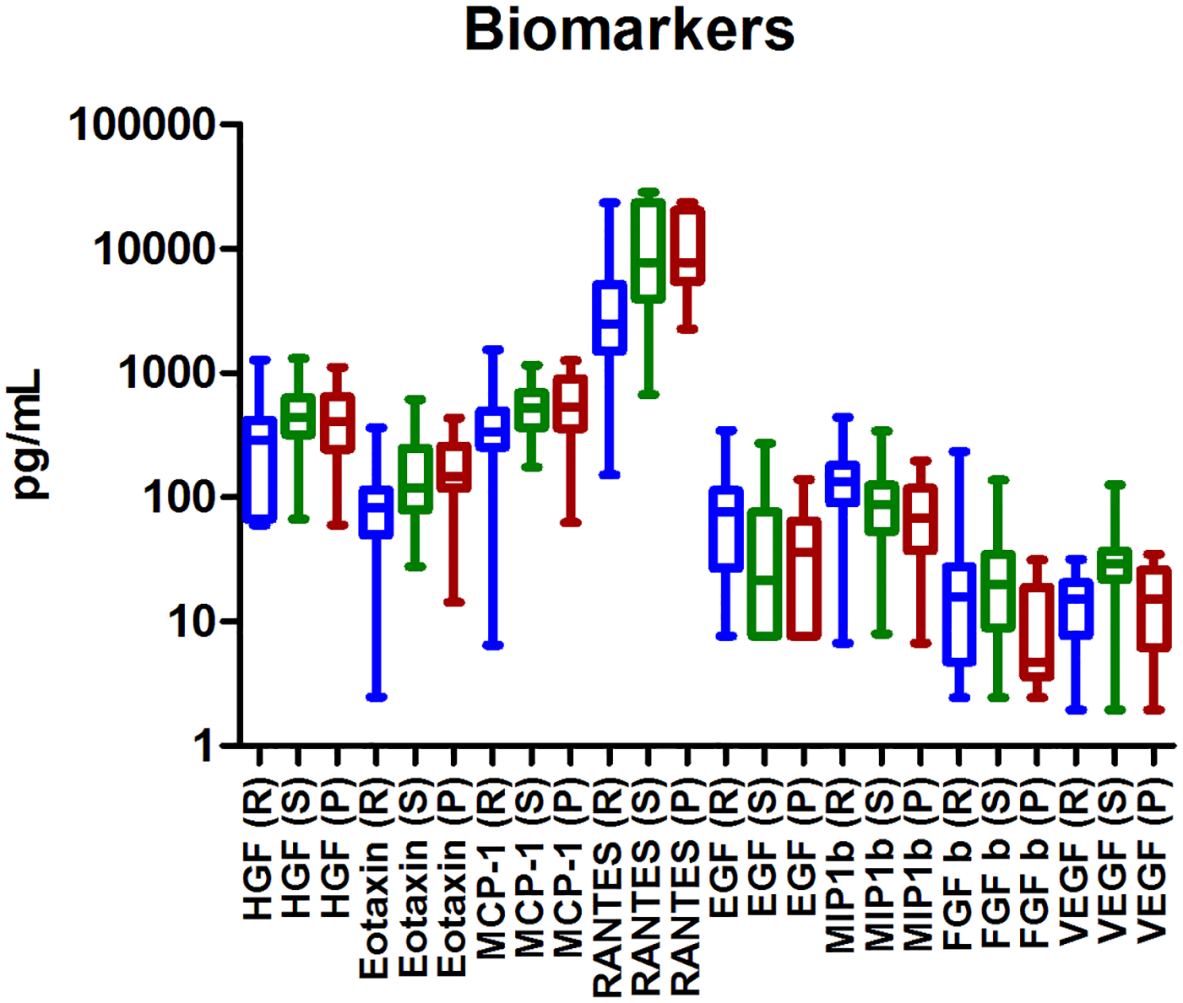
Plasma biomarkers regulations in our MS clinical forms. The combinations of biomarkers were down or up-regulated simultaneously in the individuals. The results are represented with a box and whiskers graph. The bottom and top of the box represent the first and third quartiles, the median values are represented by the line inside the box and the ends of the whiskers represent the minimum and maximum of the values. Biomarkers from Relapsing-remitting MS patients are represented with an R, secondary-progressive MS patients are represented with and S and primary-progressive MS patients are represented with a P. Note that the Relapsing-remitting group comprises the clinical groups: RR-MS Remission, RR-MS Active, RESPONDERS, NON RESPONDERS and RELAPSES.

The combinations of biomarkers were simultaneously down or up-regulated in the individuals. In Fig 6, the different concentrations of the biomarkers in each clinical form of Multiple Sclerosis patients RR-MS, SP-MS and PP-MS are represented.

## Discussion

In MS, cytokines have been thoroughly studied, but the role of chemokines and even more of growth factors in the pathogenesis of the disease remains to be elucidated. Studies analyzing comprehensive cytokines, chemokines and growth factors profiles in a large cohort of patients with MS are lacking. Here we attempt to identify relevant circulating cytokines, chemokines and growth factors associated with the diverse pathophysiological pathways that encompass MS through the comparison of a broad array of plasma biomarkers in patients with distinct clinical forms of MS.

To the best of our knowledge, we are the first to describe significant differences in circulating levels of FGFb, HGF, VEGF; Eotaxin/CCL11, MCP-1/CCL2 and Rantes/CCL5 discriminating among MS clinical forms. The biomarker analysis described herein provides a revealing cross-section of the pathophysiological MS conditions. Despite recent advances in disease-modifying therapies and biologicals for MS, current treatment regimens remain characterized by disappointment due to a failure to stop progression in PP-MS and potentially life-threatening adverse effects. There is an urge need to recognize and predict outcome in the individual MS patient that could enable more focused treatment strategies. Therefore, the identification and development of targeted therapies has moved to the forefront of the MS translational research.

Chemokines and their receptors have an important role in the pathophysiology of MS given the organ-specific nature of MS. They can act as chemoattractants through the recruitment of autoreactive cells from the periphery to the CNS and induce the secretion of proinflammatory cytokines, which promote the final demyelination and neuronal loss [11, 12]. CXCL10/IP-10, CCL2/MCP-1, CCL3/MIP-1a, CCL4/MIP-1b, CCL5/Rantes, CCL7/MCP-3 and CXCL9/Mig [11–25] are among the described proinflammatory chemokines produced by immune cells and CNS glia in MS and in the animal model of MS, experimental autoimmune encephalomyelitis (EAE). Most of the β-chemokines or CC chemokine ligands (CCL) genes are clustered in chromosome 17q11.2–12, a location that has been associated with MS in different studies [26–28]. In a model of EAE, increased levels of Eotaxin/CCL11 were associated with milder disease phenotype, tighter blood brain barrier, reduced antigenic specific response and an anti-inflammatory Th2 phenotype [29].

In addition, different growth factors have relevance in the CNS disorders. The hepatocyte growth factor (HGF) is a multifunctional protein in the CNS that is produced by microglial cells, oligodendroglial progenitor cells, astrocytes and neurons [30–33]. The receptor for HGF, c-Met, is the tyrosine kinase product of the c-Met proto-oncogene [34, 35] and both HGF and its receptor c-Met are present during brain development and persist in adulthood with important neurotrophic functions [36, 37]. Besides, peripheral HGF shows immunomodulatory effects: promoting the adhesion of B cells [38], migration of T cells [39] and recruitment of dendritic cells (DCs) [40, 41]. HGF has been found to be increased in CNS diseases, including MS and in prototypic neurodegenerative diseases, such as Alzheimer’s disease [42]. However, other studies have found decreased HGF CSF levels in patients with MS [43, 44] or unaltered levels of plasmatic HGF between MS patients and healthy controls [44]. The epidermal growth factor (EGF) has positive effects in the proliferation and differentiation of neurons, astrocytes and oligodendrocytes [45, 46] and its co-administration with growth hormone (GH) in EAE improved the clinical score and survival rate of mild and severe EAE forms [47]. In MS patients, EGF in CSF has been found to be significantly lower than in other non-inflammatory neurological diseases [48]. Also, the basic fibroblast growth factor (FGFb or FGF-2), is a key regulator of the growth, differentiation, migration and survival of CNS glial progenitors [45], and its expression is enhanced in active MS lesions [49]. FGFb has a controversial role in MS. It has been associated with demyelination and inhibition of myelin production by oligodendrocytes *in vivo* [50, 51] while with the induction of myelination in oligodendrocytes *in vitro* [52]. Recently, the knockout mouse of FGFb revealed rather a neuroprotective role for this growth factor in the animal model of MS [53].

Here we show that a low expression of FGFb/FGF-2 is a selective marker of PP-MS, probably reflecting low remyelination related to progressive neurodegeneration. FGFb is an emerging MS biomarker with a controversial role in promoting myelination by oligodendrocytes [50–52], since the knockout mouse of FGFb in EAE showed that this growth factor has a neuroprotective/regenerative role avoiding nerve fiber degeneration and axonal loss and favoring remyelination in the CNS [53]. In MS, FGFb has been found to be increased in the CSF and serum of MS patients, with the highest levels in clinically active MS patients undergoing relapse and in SP patients with disability progression [54]. FGFb is a main mitogen of oligodendrocyte precursor cells (OPCs), mainly expressed at MS periplaques where macrophagic and/or activated microglial and perivascular astrocyte-related remyelination, further supporting its role in neurorepair and neuroprotection, respectively, as well as in maintaining the integrity of the blood-brain barrier [49]. In our cohort of MS patients, we found that patients with PP-MS had significantly low levels of FGFb. In contrast, clinically active patients with SP-MS and patients at relapse showed similar levels to those of healthy controls. This finding is in agreement with the study by Sarchielli et al. who reported an elevation of FGFb in the CSF of MS patients, and the highest levels at relapse and in SP-MS patients with a recent increase in disability [54]. FGFb might be playing a compensatory role during the inflammatory attack and during the axonal insult that occurs at these stages of the disease. The fact that PP-MS patients had marked lower FGFb circulating levels may be pointing to an impaired production with respect to other MS clinical forms, suggesting an additional defect in PP-MS to restore myelination and explain their rapid disability progression compared to other patients. FGFb could be used as a future therapeutic target to induce effective migration of MS lesions by OPCs and to favor remyelination of lesions.

In our cohort of MS patients, the SP-MS form was characterized by overexpression of the vascular endothelial growth factor (VEGF) with respect to RR-MS and even to PP-MS patients. VEGF is a prominent player in the complex and highly regulated process of angiogenesis and a proinflammatory factor [55–58] suggesting its role in the RR-MS to the SP-MS transition. An increase of VEGF-A in the initial phases of relapse is compatible with its role as a pro-inflammatory factor that attracts monocytes and lymphocytes, upregulates immunomodulatory adhesion molecules, stimulates secretion of proinflammatory cytokines, and increases blood-brain barrier permeability [59, 60]. Evidence for the occurrence of neovascularization in MS has been observed by contrast-enhanced MRI in the appearance of “ring enhancement” at the periphery, but not at the center of chronic lesions [61] Another MRI study showed a direct correlation between serum VEGF levels and the magnitude of spinal cord lesions, suggesting that VEGF might be involved in the formation of MS spinal cord lesions [62]. Increased levels of VEGF and its receptor VEGFR-1 are found in astrocytes in MS plaques during the inflammatory phase [63, 64]. In addition, intrastriatal injection of VEGF aggravates plaque inflammation at the site of injection [64]. Moreover, as VEGF expression is highly influenced by inflammatory cytokines and ischemia (see below), the accumulation of VEGF may be not only a mediator but the result of MS inflammation. While all these findings may suggest that VEGF, as a factor affecting vessels or inflammatory cells, aggravates MS, VEGF, as a neuroprotective factor, can also protect against axonal damage in MS. Thus the precise role of VEGF in MS remains enigmatic. Possibly, VEGF exerts a dual role in MS lesions: increased levels of VEGF can amplify vascular permeability in vessels and thus inflammation through glial cells during the acute phase of the disease, but can also stimulate the proliferation of neurons and their axons during the chronic phases of the disease [60]. Recent evidence described low RNA VEGF levels in CSF and blood PBMCs of SP-MS patients with respect to RR-MS [65].

By our logistic regression model combining four relevant biomarkers (HGF, Eotaxin/CCL11, EGF and MIP-1β/CCL4), there was a significant overall model fit discriminating a dichotomous RR-MS versus progressive forms, with a specificity of 89.9% and a negative and positive predictive value of 82.7% and 82.5%, respectively. In our cohort of patients, having normal (similar to HC) plasma HGF and Eotaxin/CCL11 levels and low plasma EGF and MIP-1β/CCL4 levels were prognostic risk factors for being classified as a progressive patient with MS (SP-MS or PP-MS). The levels of HGF, MCP-1/CCL2 and Eotaxin/CCL11 were higher in SP-MS and PP-MS patients than in RR-MS, but similar to those observed in healthy controls. Therefore, at the RR-MS stage of the disease, circulating levels of the β-chemokines MCP-1/CCL2, Eotaxin/CCL11 and of the growth factor HGF, were diminished with respect to other MS clinical forms and to healthy controls, suggesting a potential role for these diminished molecules in the initial stages of MS pathogenesis. MCP-1/CCL2 levels have been reported to be decreased in MS patients [11, 66–69] although its expression in MS lesions is increased [28]. MCP-1/CCL2 stimulates in vitro the production of HGF by a mouse macrophage cell line [70] and in vivo Müller et al. reported that low levels of MCP-1/CCL2 correlated strongly with low levels of HGF at the central compartment [44]. We have found that plasma levels of the β-chemokines: MCP-1/CCL2, Rantes/CCL5 and Eotaxin/CCL11 correlate strongly with plasma levels of HGF in MS, suggesting a physiopathological link between HGF and these β-chemokines. The levels of Rantes/CCL5 were markedly elevated in the progressive forms with respect to healthy controls and to RR-MS patients. Thus, high circulating levels of Rantes/CCL5 might be reflecting a more progressive and disabling disease course. Rantes/CCL5 and its receptor CCR1, CCR3 and CCR5 have been detected in active demyelinating plaques on immune and microglia cells [71, 72], its expression in serum is higher in RR-MS patients with active lesions and clinical activity [68, 73, 74] and its concentration decreases in CSF after corticosteroid therapy [75]. Moreover, circulating T lymphocytes of progressive MS patients express higher expression of the Rantes/CCL5 receptor CCR5 than those from healthy controls and show an increased migration towards Mip-1α/CCL3 and Rantes/CCL5 with a skewed Th1 phenotype [76, 77].

In addition, the levels of HGF, MCP-1/CCL2, Eotaxin/CCL11 and Rantes/CCL5 that were all overexpressed in progressive MS patients correlated strongly with EDSS, the classical indicator of clinical disability in MS, while EGF and MIP-1β/CCL4 that were diminished in progressive patients inversely correlated with EDSS.

On the other hand, we found that the plasma levels of EGF and of the β-chemokine MIP-1β/CCL4 were lower in SP-MS and PP-MS clinical forms with respect to RR-MS patients and to healthy controls. Scalabrino et al. found lower levels of CSF EGF in RR-MS and SP-MS patients with respect to other non-inflammatory neurological diseases, but they found no differences in serum EGF among MS clinical forms [48], in this study the sample size was very limited. In the present study, we do have found significantly diminished plasma EGF levels in progressive MS patients than in RR-MS and than in healthy controls. MIP-1β/CCL4 shares the same chemokine receptor, CCR5, with Rantes/CCL5 and with MIP-1α/CCL3. The target cells of MIP-1β/CCL4 are CD8^+^ T lymphocytes and it has been detected in actively demyelinating plaques where it is expressed by macrophages and microglia [12, 62].

The high degree of biological heterogeneity that characterizes MS, wherein the deregulated inflammatory and neurodegenerative pathways vary, has hindered the clinical impact of targeted therapies and emphasizes the need for improved tools aimed at identifying those patients most likely to benefit from a particular treatment.

IP10/CXCL10 and MCP-1/CCL2 have previously been reported to be at higher levels in patients under treatment with IFN-β [78–81] and have been proposed as biomarkers for IFN-β response [79, 80]. In this work, we observed that long term treatment with IFN-β translates into persistent IP10 plasma levels in these patients, with values significantly higher than those found in healthy controls and in the rest of groups with RR-MS, including RR-MS patients that had not previously responded to IFN-β therapy. In addition, we observed that MCP-1/CCL2 discriminated between responder and non responder patients to IFN-β therapy. Moreover, we observed that SP-MS patients had significantly higher circulating levels of IP10 than patients in a RR-MS stage (excluding those patients with RR-MS but under therapy with IFN-β) and than healthy controls. IP10 is a chemokine expressed by astrocytes that is upregulated and highly expressed in active demyelinating lesions [12, 66]. This chemokine has been found at increased levels in serum and in the CSF of MS patients during exacerbations [66]. However, in our cohort of patients we did not observe higher plasma levels of IP10 in MS patients at relapse. Interestingly, we remarked that progressive patients, especially SP-MS had increased circulating levels of IP10. Several studies have reported increased endogenous production of IFN-γ by activated T lymphocytes expressing CCR5 from progressive MS patients [82, 83]. As IP10 is induced by IFN, a high endogenous production of the Th1 cytokine, IFN-γ, by Th1 CCR5^+^ lymphocytes might be reflecting a positive endogenous loop of IP10 production.

**Fig 7:**
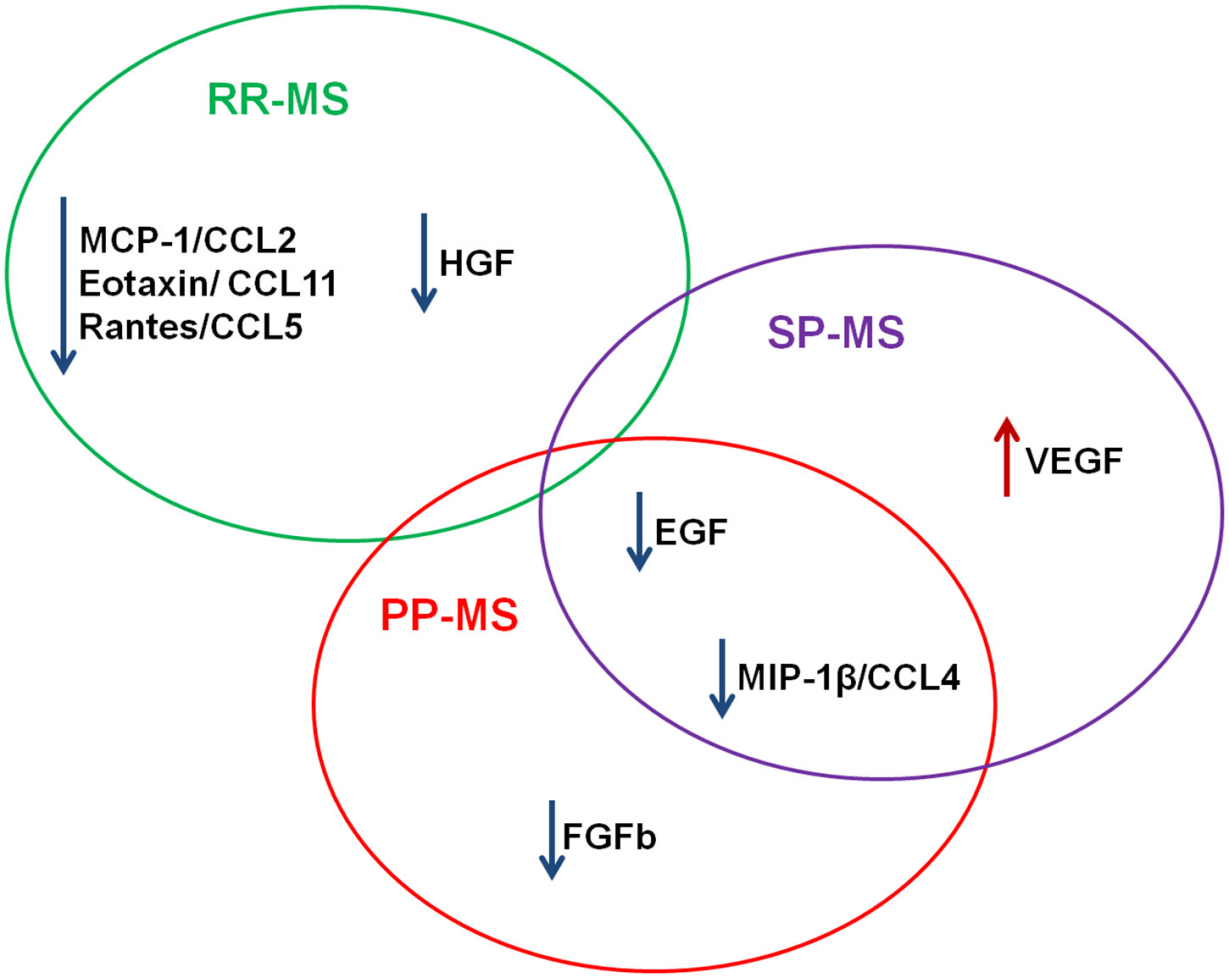
Plasma biomarkers significant across our MS clinical forms. Findings are summarized. Listed biomarkers were found to differ significantly between comparison groups. Arrow preceding each biomarker name indicates increase or decreased plasma concentrations in MS groups.

This investigation illustrates the unique and informative role of plasma profiling in advancing our understanding of diverse pathophysiological pathways underlying the different MS clinical forms and response to treatments. The plasma biomarkers significant across MS clinical forms are summarized in Fig 7. There are scarce studies on the spatial and temporal differential expression of the chemokine system in the clinical forms of MS. The fact that among a combination of 30 human cytokines, chemokines and growth factors; different plasma expression among clinical groups was only evident for chemokines and growth factors, underlines the importance of these proteins in the clinical course and pathophysiology of an organ-specific autoimmune disease, such as MS. Autoreactive immune cells have a CNS tropism and migrate to their target organ, attracted by chemokines either through direct chemoattraction or by the activation of their leukocyte integrins [84, 85]. Growth factors have outstanding relevance peripherally and also in the central compartment, where they can participate in neovascularization and remyelination to counteract the inflammatory attacks. Our findings suggest that factors involved in the chemokine inflammatory response and growth/angiogenic factors may differentially account for divergent MS clinical course. It is crucial that ongoing work in the field of non-invasive biomarkers will be aimed at pinpointing the origins and functional roles of the identified biomarkers, which could also allow more focused application of different therapies. Continued investigation into the relationships between these factors should reveal new insights into the complex mechanisms underlying MS pathophysiology.

We carried out an evaluation of circulating biomarkers in order to identify specific biomarkers and combinations of them which might serve as effective tools in the diagnosis and therapeutic targeting of MS patients. The biomarkers and their combinations found in this work might be a useful diagnostic tool for a more accurate classification of MS clinical forms and thus prompt treatment for those patients with progressive MS, for whom there is currently no therapy available.
